# Scale, mergers and efficiency: the case of Dutch housing corporations

**DOI:** 10.1007/s10901-016-9515-4

**Published:** 2016-05-19

**Authors:** Jacob Veenstra, Hendrik M. Koolma, Maarten A. Allers

**Affiliations:** 1 Faculty of Economics and Business, Centre for Research on Local Government Economics, University of Groningen, PO Box 800, 9700 AV Groningen, The Netherlands; 2 Faculty of Social Science, VU University Amsterdam, De Boelelaan 1105, 1081 HV Amsterdam, The Netherlands

**Keywords:** Social housing, Housing corporations, Efficiency, Mergers, Data envelopment analysis, Stochastic frontier analysis

## Abstract

The efficiency of social housing providers is a contentious issue. In the Netherlands, there is a widespread belief that housing corporations have substantial potential for efficiency improvements. A related question is whether scale influences efficiency, since recent decades have shown a trend of mergers among corporations. This paper offers a framework to assess the effects of scale and mergers on the efficiency of Dutch housing corporations by using both a data envelopment analysis and a stochastic frontier analysis, using panel data for 2001–2012. The results indicate that most housing corporations operate under diseconomies of scale, implying that merging would be undesirable in most cases. However, merging may have beneficial effects on pure technical efficiency as it forces organizations to reconsider existing practices. A data envelopment analysis indeed confirms this hypothesis, but these results cannot be replicated by a stochastic frontier analysis, meaning that the evidence for this effect is not robust.

## Introduction

In the Netherlands, social housing is provided by housing corporations^1^, privately owned non-profit organizations executing a public task. Dutch housing corporations own over 70 percent of all rental housing, which boils down to one-third of the total housing stock.^2^

Currently, the Dutch corporation sector is in the spotlights due to various incidents, ranging from integrity violations to billions of euros lost on high-risk projects and financial derivatives. These incidents led Parliament to start-up an inquiry in 2013 which concluded, among other issues, that Dutch government has failed to establish effective control of the efficiency of the housing corporations (Parlementaire Enquêtecommissie Woningcorporaties 2014).

There are several reasons to suspect that housing corporation efficiency is not optimal. The Dutch government withdrew from active involvement in the 1990s, which greatly enhanced the autonomy of corporations. The resulting lack of governmental oversight, combined with weak competition and loose corporate governance, allowed housing corporations considerable operational leeway (Parlementaire Enquêtecommissie Woningcorporaties 2014). Moreover, housing corporations are not allowed to appropriate profits, which further weakens the incentive to operate efficiently (Walker and Murie 2007). Finally, many corporations enjoyed a relatively wealthy position and were able to increase revenues through sale of formerly subsidized dwellings.

Parlementaire Enquêtecommissie Woningcorporaties (2014) suggests that the current institutional design should be reconsidered because it gives too much occasion for inappropriate behaviour. However, Priemus (2003) pointed out that one cannot justify any kind of reform in the social housing sector because in the current situation ‘we are under-informed about the efficiency of housing corporations’ (p. 269). Clearly, there is a need for a coherent measurement of the efficiency of corporations. This paper attempts to fill this hiatus.

An important follow-up question is whether there exists a relationship between the scale of operations and efficiency. The last decades have seen many mergers of housing corporations, and more are to be expected. The effects on efficiency are far from clear, however. In many public service sectors, the scale of operations is an important point of discussion, considering the vast literature on this issue (see, e.g. Holzer et al. 2009; Leithwood and Jantzi 2009; Blank et al. 2011).

The fact that mergers are not always driven by efficiency considerations is illustrated by the existence of many alternative merger motivations that have been put forward: herding (Scharfstein and Stein 1990; Devenow and Welch 1996), hubris (Roll 1986), entrenchment (Shleifer and Vishny 1989), empire building (Rhoades 1983) and institutional isomorphism (DiMaggio and Powell 1983). Research confirms that within the Dutch corporation sector, only few mergers were conducted out of efficiency considerations (Sect. 3.2).

In theory, the effect of merging on efficiency is ambiguous. In principle, according to Bogetoft and Wang (2005), a merger can be beneficial (or detrimental) for three reasons. First, a merger increases scale. If the production technology is characterized by economies of scale, increasing scale would improve efficiency. On the other hand, if there are diseconomies of scale, a merger will have a negative effect. Bogetoft and Wang (2005) call this the ‘scaling or size effect’. If organizations operate under economies of scale, increasing scale will reduce average costs because fixed costs are spread over a larger output, and because of specialization due to a better division of labour (economies of scale). On the other hand, if organizations grow too large, diseconomies of scale may set in due to increased internal complexity and weaker connections with customers. As a result, the unit cost of (public) services is often assumed to be u-shaped, reflecting economies of scale (downward sloping average expenditures) for units below a certain critical size and diseconomies of scale for larger organizations.

Secondly, a merger might lead to a reconsideration of business practices because a new management team is brought in, or because the organizations learn from each other’s practices. Existing organizations usually have well-established ways of doing things, even though more efficient practices have become available (technological progress). A merger, bringing together organizations used to doing things in different ways, forces them to reconsider procedures and operations and gives an opportunity to learn from each other. This may result in the adoption of more efficient practices (see also Hansen et al. 2014).^3^ For the remainder of this paper, we label this reasoning as the ‘shake-up hypothesis’.

Thirdly, a merger combines two sets of inputs and outputs into one set. It might be that the mixture of this new set is more favourable (i.e. more balanced) than the original sets. Bogetoft and Wang (2005) call this the ‘harmony, scope or mixture effect’.

In this paper, the main question we try to answer is: What are the effects of scale increases and mergers on both scale efficiency and pure technical efficiency? This paper offers a framework to assess the operational efficiency of housing corporations, and to analyse the efficiency effects of increasing or decreasing scale by means of both a data envelopment analysis (DEA) and a stochastic frontier analysis (SFA).

## Institutional context and recent developments

### The Dutch institutional setting

Many countries provide subsidized housing to low-income households. Although in the Netherlands, corporations do not receive subsidies any more, they do have the advantages of a favourable financial position as a result of subsidies received in the past, and of low financing costs because of a bail-out scheme that guarantees corporations’ loans. In the Netherlands, the social housing sector is especially large (Smith and Oxley 2007; Whitehead and Scanlon 2007). In 2012, there were 381 housing corporations, owning 2.2 million dwellings.

As private institutions facing the statutory obligation to execute public tasks, Dutch housing corporations are hybrid organizations (Blessing 2012). The most salient consequence of their legal structure is the absence of owners, shareholders or influential stakeholders. Ruled by public law, housing corporations are prohibited to distribute profit (‘non-distribution constraint’). The corporate governance structure resembles the principal-agency model (Jensen and Meckling 1976), but the absence of owners allows wealth sharing by managers and members of the organization (Jensen 2000). Unlike charitable non-profits, Dutch housing corporations are neither donor-financed nor driven by volunteers. They may be characterized most appropriately as non-profit enterprises (Anheier and Ben-Ner 2003): professionalized private corporations with a public purpose, and without residual claimants. The absence of a profit-maximizing objective may weaken incentives to maximize efficiency (Walker and Murie 2007).

Also, the ties between government and corporations are weak, both financially and operationally. Indeed, in 1995, housing subsidies ceased to exist, since the balance of outstanding government loans and the present value of future subsidy obligations was paid out as lump sums. This enhanced the autonomy of corporations and introduced cash windfalls in the sector (Koolma 2008). Operationally, the only binding condition that has to be fulfilled is that housing corporations must use all of their resources for (activities strongly related to) public housing. Additionally, the government has formulated a set of ‘performance fields’ by means of the Social Housing Management Decree (*Besluit Beheer Sociale Huursector*, BBSH; see Box 1). However, corporations can freely determine which tasks to give priority. There has not been an effective system to check whether any of these goals are reached.^4^

**Box 1:** BBSH performance fields

1. Adequate housing of the target group, that is, households with relatively low income.
2. Preserving the quality of the housing stock.
3. Improving livability of neighbourhoods.
4. Providing housing and fostering services to the elderly, the disabled or other persons that are in need of care or guidance.
5. Preserving financial continuity.
6. Enabling renters to get involved with corporation policy and administration.
7. Operating efficiently.

Another reason why efficiency may not be optimal is lack of competition. In the Netherlands, the bulk of social housing is in the hands of housing corporations. There is almost no market sharing with commercial or cooperative organizations. Because of exploitation schemes where cash flows are negative for the first 10 years after construction, entry of new housing corporations is almost impossible (Koolma 2008, p. 356). Competition is further weakened by the regional concentration of the housing stock of the different corporations.

### Mergers

**Fig. 1:**
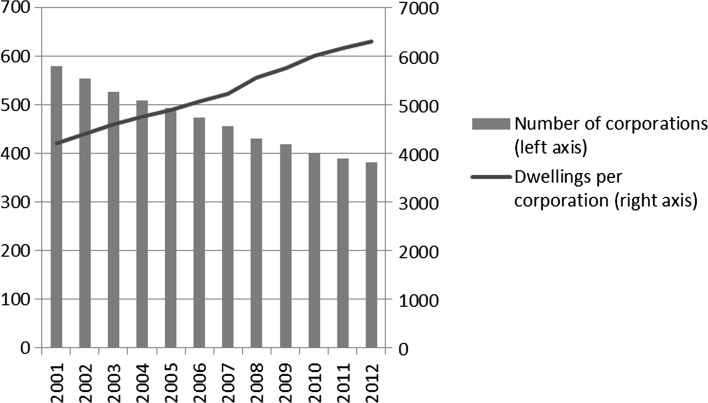
Total number of housing corporations, and dwellings per corporation, 2001–2012

As noted, the last decades have shown a boom in merger activity among corporations. As a result, the total number of corporations declined from to 858 in 1985 to 381 in 2012.^5^ Because the total housing stock of corporations remained fairly constant, the number of dwellings per corporation increased sharply. Figure 1 illustrates this for 2001–2012.

## Previous empirical research

### Efficiency

Empirical research on the efficiency of Dutch housing corporations is scarce. Koolma (2008) presents a set of general findings that support the notion that efficiency in the Dutch social housing sector could be enhanced. For example, there is large variation in cost levels and goal achievement between corporations. Also, a random sample of 25 percent of corporation annual reports in 2002 showed that only two percent of them mentioned the concept of cost reduction explicitly. This suggests that efficiency might not have been a major issue in that period.

The only attempt to measure efficiency of housing corporations we are aware of is De Graaf et al. (2001), who use a data envelopment analysis on a subset of housing corporations in 1998. The authors conclude that the efficiency potential in the sector is low. The researchers acknowledge, however, that these results should be interpreted with caution. Their method of data revision and processing reduces the dataset to only ten percent of the population.

Our approach differs in five ways from the research of De Graaf et al. (2001). First, we use a broad panel dataset instead of a cross section, so that the change in productivity can be assessed. Secondly, our method of combining other data sources with the dataset of corporations leaves the entire population of corporations intact.^6^ Thirdly, we use different output measures. Fourth, we add to this the explicit study of the relation between scale and efficiency. Finally, we use both a data envelopment analysis and a stochastic frontier analysis.

### Scale and mergers

Merger motives for housing corporations are quite heterogeneous: improving market position (Van Veghel 1999; Cebeon 2006; Koolma 2008), increasing professionalism (Van Veghel 1999), improving efficiency (Cebeon 2006; Koolma 2008) or resolving financial problems (Koolma 2008; Veenstra et al. 2013). Only a minority of the mergers was explicitly motivated by taking advantage of scale economies (Van Bortel et al. 2010). This confirms the notion that for Dutch corporations, efficiency has long not been recognized as a major issue. For English housing associations on the other hand, efficiency appeared to be a more important motive (Van Bortel et al. 2010).

Studies on the effects of scale increases and mergers do not find conclusive evidence. Based on a cross section of housing corporations in 2002, Koolma (2008) finds evidence suggesting that larger corporations face higher costs than their smaller counterparts, whereas there is only a weak effect on the scope of their portfolio management and no effect on the level of investments. This suggests that many corporations operate at diseconomies of scale. Van den Berge et al. (2013) confirm this by noting that merged corporations have higher average costs than corporations that did not merge. However, according to the authors this cannot be ascribed to the merger itself since merging does not lead to an increase in costs. In another recent study, Crooijmans (2015) investigates the relation between mergers and several measures that serve as proxies for productive efficiency and finds hardly any significant relationships.

Mullins (2006) indicates that, within the English social housing market, there is a belief that efficiency gains from increasing scale (and merging) can be obtained. Not all English housing associations agree on this, however (Mullins 2007). Lupton and Kent-Smith (2012) argue that there is hardly any relation between costs and scale of English housing associations and that the effects of mergers are ambiguous as well. However, a few case studies investigated in Lupton and Kent-Smith (2012) indicate that mergers can be successful, but this success is most probably caused by the merger changing internal processes instead of a scale effect. This means there may be a shake-up effect. A merger therefore does not automatically improve performance. The question is thus whether the efficiency gains could also have been realized without the merger. That is, is it the scale increase that gave rise to the efficiency gains, or is it the organizational change and increased focus on efficiency, or both?

## Methodology

In a sector with a large number of decision making units (dmu’s), relative efficiency can be measured by comparing the input–output mix of a certain dmu with that of (all) other dmu’s. In the literature, frontier analysis is the most frequently applied method. Frontier analyses can be both nonparametric, e.g. data envelopment analysis (DEA, Farrell 1957; Charnes et al. 1978) and parametric, e.g. stochastic frontier analysis (SFA, Aigner et al. 1977; Meeusen and Van den Broeck 1977). Both parametric and nonparametric methods construct a best practice frontier based on the data. DEA constructs this frontier by means of linear programming, while SFA estimates the frontier econometrically. Which method is most appropriate depends on the setting.

The main advantage of DEA is that one does not need to specify a functional form of a production function, which is required for SFA. As Pestieau (2009) notes, DEA needs only a few weak assumptions (free disposability, and the choice between convexity and proportionality in returns to scale). The major disadvantage of DEA is that it fails to account for noise in the data. Therefore, the impact of outliers in the dataset on the results might be considerable. Also, Simar and Wilson (2013) argue that using DEA-scores for making inferences (i.e. using DEA-scores in regression analysis) is difficult and prone to incorrect estimations since it does not describe the data-generating process in a coherent way. Because the production function is hard to identify and we do not have data on input prices, we will first use DEA. As a robustness check, we will also use SFA.

### Data envelopment analysis

**Fig. 2:**
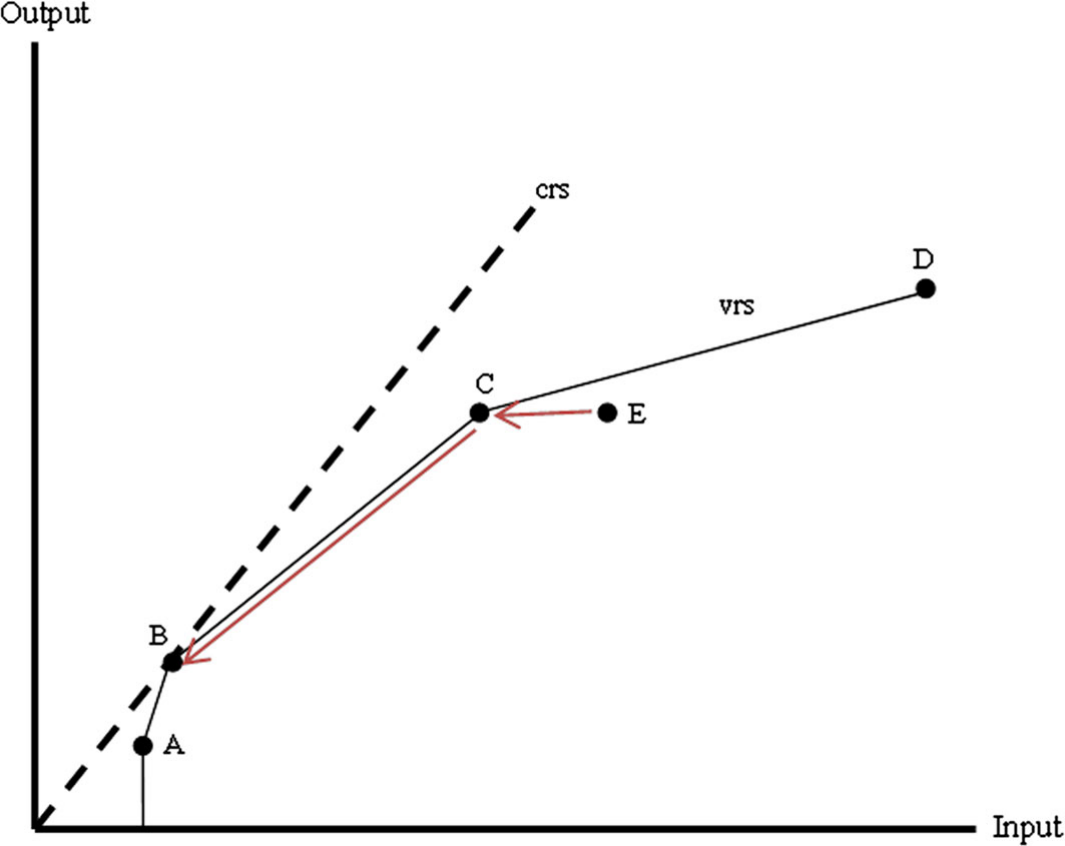
DEA with 1 input and 1 output, crs- versus vrs-specification

We discuss DEA by means of a simple example. Technical details are presented in the “Appendix”. Figure 2 provides a case with five dmu’s, one input and one output. Dmu B has the highest output/input ratio, and therefore the highest productivity, so it is located on the constant returns to scale (crs) frontier (the dashed line). The crs-frontier assumes that the relation between inputs and outputs is linear. As Geys and Moesen (2009) note: ‘Such an assumption may be valid over limited ranges of production, but is unlikely to be justifiable in general’ (p. 7). Therefore, we may introduce a frontier that assumes a variable returns to scale (vrs) technology. This is represented by the solid line in Fig. 2.

We may now distinguish between three definitions of efficiency. A dmu located on the vrs-frontier is *pure technically efficient*, meaning that given the current scale of operations, it cannot improve its efficiency. This holds for dmu’s A, B, C and D in Fig. 2. However, B is the only dmu that has a maximum *scale efficiency* as well, because it is located on the crs-frontier. This means that given the current technological possibilities, no dmu is more productive than B. Therefore, the distance to the crs-frontier measures the *total (technical) efficiency.* Total technical efficiency (TE^crs^) is thus the product of pure technical efficiency (TE^vrs^) and scale efficiency (SE): TE^crs^ = TE^vrs^ * SE. In Fig. 2, dmu A operates at economies of scale as it is smaller than the optimal scale level at B. Similarly, C and D operate at diseconomies of scale.

### Non-discretionary inputs and exogenous variables

Some inputs may be non-discretionary or fixed. These inputs are relevant factors in the production process in year *t* but cannot be influenced anymore during the production process (see Banker and Morey 1986). In the case of housing corporations, the housing stock at the beginning of the year should be included as a fixed input (see also Sect. 5.1).

Closely related to the concept of fixed inputs is the idea that there may exist exogenous variables that influence efficiency scores.^7^ For example, suppose that dmu A is (dis)advantaged because of exogenous circumstances. To account for this, we can include the restriction that this dmu should only be compared with other dmu’s that are not exogenously advantaged relative to dmu A (Ruggiero 1998). In other words, advantaged dmu’s are being removed from the best practice frontier.

### Malmquist indices

To calculate efficiency changes over time, we use Malmquist indices (see Färe et al. 1994; Coelli 1996). To clarify this, we give a numerical example (for a general explanation, see the “Appendix”). Suppose dmu A has a crs-efficiency score of 0.6 in year *t*_0_. Now, suppose we were to take the input–output mix of dmu A in year *t*_1_, but keep the frontier fixed. If the efficiency score has increased to 0.75, we can ‘safely’ interpret this as an increase in productivity of (0.75–0.60)/0.60 = 25 %, because we have used the same frontier (that of *t*_0_) as before. Alternatively, we could use the frontier of *t*_1_ both times just as well. If this would yield a productivity increase of 30 percent, the Malmquist index becomes √(1.25*1.30) ≈ 1.27. This indicates that total factor productivity change is 27 percent.

*Total factor productivity change* can be decomposed into a *change in technology* (i.e. the total shift of the crs-frontier over time) and the *change in efficiency* (the extent to which a dmu approaches the crs-frontier) (Coelli 1996). The change in efficiency can be decomposed further into *pure technical efficiency change* (approaching the vrs-frontier) and *scale efficiency change*. In the example of Fig. 2, a movement from E to C resembles pure technical efficiency change and a movement from C to B indicates an increase in scale efficiency.^8^

### Mergers and efficiency in a nonparametric setting

As noted, a merger may influence efficiency via (1) a scale effect, (2) an effect on pure technical efficiency and (3) a mixture effect. In this paper, we will ignore potential mixture effects of mergers and thus focus on the effects on scale efficiency and pure technical efficiency. Since we use only one input in our model, mixture gains could only be achieved by mixing of outputs. Since corporations are single-purpose entities, we assume that potential gains from mixing are negligible. Moreover, to the best of our knowledge, current software does not allow for a straightforward implementation of mixture effects. In the next section, our approach is presented.

### Stochastic frontier analysis

As noted, DEA-results are sensitive to data outliers and using DEA-scores for making inferences may be problematic. Therefore, we use SFA as a robustness check. SFA constructs the best practice frontier econometrically by estimating a production or cost function. Efficiency is then determined by decomposing the error term into a traditional white noise term (*v*) and an inefficiency term (*u*).

Just as under DEA, SFA allows us to construct measures of total productivity change and decompositions into (1) pure technical efficiency change, (2) a scale effect and (3) technological change (see the “Appendix” for calculations).

In the next section, our model specifications are presented.

## Models and data

### Models

Measuring efficiency of organizations in the public sphere faces several obstacles (Stevens 2005; Veenstra et al. 2013). The main question is: What exactly are the inputs and outputs of the production process? It is especially difficult to distinguish between output (the direct activities of the organizations) and outcome (the benefit to society as a result of the activities). Another question is whether data availability is sufficient. In general, we argue that an output should meet three requirements: it should be (1) measured (i.e. data are available), (2) it should be influenced by input, (3) it should add to social welfare. In this paper, we use a basic model ensuring data availability over multiple years: it includes as outputs the total number of new housing allotments, the number of continued rent contracts and the increase in housing quality.^9^ New allotments have been split up into four outputs: (1) persons below 65, housed adequately, (2) persons below 65, housed inadequately, (3) elderly housed adequately, (4) elderly housed inadequately. Adequate housing in this context means providing a dwelling that has a rent that fits the tenant’s income (neither too high nor too low). It does not refer to adequacy in terms of physical condition of the dwelling, or appropriateness for household size. The distinction between the categories is made since adequate housing may result in higher search costs.

The number of continued contracts is broken down into households in (1) special dwellings suitable for the elderly and disabled and (2) all other dwellings. Tenants in special dwellings may demand more time and energy from the corporation staff because they need more services than others.^10^

Housing quality is measured by means of the so-called Housing Valuation Scheme (*Woningwaarderingsstelsel*, WWS). This assigns points to every dwelling on the basis of physical characteristics, such as size and type of dwelling, sanitary fittings, energy efficiency, etc. Because we use both vrs- and crs-specifications, a relative output measure like the average housing quality would be inappropriate (Podinovski 2004). Instead, we use the increase in average housing quality, multiplied by the weighted number of dwellings in the current year as output.

As input we use operational costs which consist of (1) wages and salaries, (2) maintenance costs and (3) other operational costs. Including capital expenditures as inputs would not alter our conclusions, however (details not shown). The number of dwellings at the start of the year is included as a non-discretionary input. Finally, average age of the housing stock, soil quality^11^ of the region where the corporation is active, and address density are included as exogenous variables. A simple regression indicates that corporations with an older housing stock, a less firm soil and a lower address density are disadvantaged. The model specification is presented in Table 1. Descriptive statistics are given in Table 2.

**Table 1:** Model specification DEA

Output	Adequate allotments (age < 65)
	Inadequate allotments (age < 65)
	Adequate allotments (age > 65)
	Inadequate allotments (age > 65)
	Continued contracts (ordinary dwellings)
	Continued contracts (special dwellings)
	Change in housing quality
Input	Operational costs
Fixed input	Number of dwellings at the beginning of the year
Exogenous variables	Average age of dwellings
	Soil quality
	Address density

**Table 2:** Descriptive statistics of housing corporations

	Average	SD	Min	Max
Adequate allotments (<65)	375	676	0	5229
Inadequate allotments (<65)	48	120	0	716
Adequate allotments (>65)	60	110	0	1604
Inadequate allotments (>65)	11	33	0	479
Continued contracts (ordinary dwellings)	4830	9080	0	55,316
Continued contracts (special dwellings)	739	1517	0	22,843
Average housing quality	135	13	80	177
Wages and salaries^a^	3310	6687	0	41,116
Maintenance costs^a^	6885	12,199	38	70,257
Other operational costs^a^	3840	8421	0	58,078
Capital expenditures^a,b^	7700	16,867	0	119,190
Average age of dwellings (years)	33	7	4	62
Address density^c^	1472	1086	174	6059
Soil quality^d^	1.10	0.15	1	1.76

^a^Amounts are expressed in thousands of 2012 euros

^b^Available for 2002–2010 only

^c^Address density measures the average number of addresses within a radius of 1 kilometre of each address

^d^A higher value means less favourable soil conditions

Outliers have been identified by calculating superefficiency scores. The superefficiency of dmu *i* is defined as the efficiency which is found after removing dmu *i* from the frontier. In this way, the efficiency score may exceed 1. We have removed all corporations with an initial superefficiency score of 3 or higher from our data.^12^

The model used for the stochastic frontier analysis is slightly other than the one for the data envelopment analysis. We use only three outputs (new allotments, continued contracts and housing quality) in order to avoid multicollinearity among regressors. The specification is given in Table 3.

**Table 3:** Model specification SFA

	Variable name
Cost variable	
C	Operational costs
Output	
Y1	New allotments
Y2	Continued contracts
Y3	Housing quality (WWS-points)
Exogenous variables	
Z1	Average age of dwellings
Z2	Soil quality
Z3	Address density

### Data

The *Central Public Housing Fund* (CFV) has provided us with a dataset that comprises all corporations between 2001 and 2010. For 2011 and 2012, we make use of publicly available data from the Central Public Housing Fund. Municipal data used as control variables are obtained from Statistics Netherlands.

## Results

### Efficiency scores and Malmquist indices

Table 4 reveals average efficiency scores of 0.74 (crs) and 0.86 (vrs). About half of the corporations are located on the vrs-frontier. Average scale efficiency (i.e. crs-efficiency/vrs-efficiency) is 0.85. This implies that the savings potential by increasing pure technical efficiency is roughly equal to the potential efficiency gains by changing scale.

**Table 4:** Static DEA-results (all years)

	Period	Average efficiency	Standard deviation	Percentage with maximum efficiency	Minimum efficiency
Total efficiency	2002–2012	0.74	0.20	25	0.24
Pure technical efficiency	2002–2012	0.86	0.17	50	0.26
Scale efficiency	2002–2012	0.85	0.16	25	0.36

N runs from 461 in 2002 to 319 in 2012

Table 5 presents scale (dis)economies in 2012. In 2012, 7 percent of the corporations operated under economies of scale, while 63 percent had diseconomies of scale. The bulk of the corporations should therefore be able to improve scale efficiency by reducing their size. Scale efficiency is highest for corporations with 501–1000 dwellings. For corporations with more than 2500 dwellings, diseconomies of scale become more prevalent.^13^

**Table 5:** Average scale efficiency in 2012 (based on DEA-results)

Number of dwellings	Number of corporations	Average scale efficiency	Percentage corporations with economies of scale	Percentage corporations with scale neutrality	Percentage corporations with diseconomies of scale
<= 500	30	0.97	37	63	0
501–1000	29	0.995	14	72	14
1001–2500	81	0.96	7	36	57
2501–5000	61	0.92	2	23	75
5001–10,000	67	0.85	0	13	87
>10,000	59	0.75	0	12	88
All corporations	327	0.90	7	30	63

In 2012, the total number of corporations was 381. Due to data omissions, this dataset comprises 327 corporations

To investigate efficiency changes over time, bootstrapped Malmquist indices are presented in Table 6.^14^ An index above (below) one indicates an increase (decrease) in efficiency. The index of total factor productivity change is decomposed into pure efficiency change, technological change and scale efficiency change (see the “Appendix” for details). We may compare these factors for both merged and unmerged corporations to see whether there is a structural difference between the two groups. Table 6 indicates that in most years, the change in pure efficiency is higher for merged corporations than for unmerged corporations. This gives some evidence in support of the shake-up hypothesis. On the other hand, each year, merged corporations have a lower scale effect, meaning that mergers often lead to, or increase, diseconomies of scale. Finally, it seems that from 2009 onwards, a trend of increasing productivity has set in. This may be a consequence of the increased attention that the subject of efficiency has received in recent years (Nieboer and Gruis 2016).

**Table 6:** TFP-decompositions under DEA (based on bootstrapped Malmquist indices)

Period	Pure efficiency change	Technological change	Scale effect	Total factor productivity change
Merged corporations				
2002/2003	1.3072	0.8217	0.7774	0.8425
2003/2004	1.2332	0.9527	0.8862	0.8913
2004/2005	1.0944	0.9777	0.8565	0.854
2005/2006	1.235	1.0459	1.1128	1.1263
2006/2007	1.2051	0.9266	1.0336	0.9104
2007/2008	1.1336	1.1144	0.8975	1.0041
2008/2009	0.907	1.1115	0.9267	0.935
2009/2010	1.2931	0.9168	0.9532	1.0266
2010/2011	1.1796	1.3259	0.9298	1.4236
2011/2012	1.4058	0.7621	0.9502	0.9957
Unmerged corporations				
2002/2003	1.2074	0.8553	1.0217	1.0275
2003/2004	1.1321	0.9912	1.1526	1.0842
2004/2005	1.0777	0.9828	0.8928	0.9716
2005/2006	1.1203	1.0227	1.2179	1.0544
2006/2007	1.1485	0.9352	1.255	1.0172
2007/2008	1.0059	1.0222	1.046	0.9946
2008/2009	0.9404	1.0995	0.9736	1.0122
2009/2010	1.3218	0.8765	1.0433	1.1116
2010/2011	0.9698	1.2746	0.9912	1.1927
2011/2012	1.4598	0.769	1.0485	1.1131

Note, however, that the Malmquist indices show peaks and dips that may seem unreasonably strong. This is why we will also conduct a parametric approach to test the robustness of these numbers (see Sect. 6.4).

### Relation between scale, mergers and efficiency: baseline results

To test the shake-up hypothesis, we estimate a regression with the Malmquist components as dependent variables. We relate scale increases and mergers to both total (crs-)efficiency and pure technical (vrs-)efficiency. The “Appendix” to this paper provides details. Table 7 presents results. The first column gives the effects of a change in the number of dwellings and of merger activity on total efficiency change (see Eq. 9a in the “Appendix”). The second column gives the effect on pure technical efficiency change (Eq. 9b).^15^

**Table 7:** Regressions of DEA-efficiency measures on scale and mergers

	(1)	(2)	(3)	(4)	(5)	(6)
	Total efficiency	Pure technical efficiency	Total efficiency	Pure technical efficiency	Total efficiency	Pure technical efficiency
Dwellings organic year *t* (*1000)	0.1070***	0.1053***	0.0976***	0.0879***	−0.0993***	−0.0196
	(3.8003)	(4.1804)	(3.6131)	(3.9680)	(−2.6371)	(−0.5792)
Dwellings organic year *t* − *1* (*1000)	−0.1355***	−0.0744***	−0.1206***	−0.0515***		
	(−5.5745)	(−3.3633)	(−5.3609)	(−2.6110)		
Dwellings merger (*1000)	−0.0016	0.0131***	−0.0022	0.0137***	−0.0080	0.0168**
	(−0.4280)	(3.0089)	(−0.5638)	(3.0642)	(−0.9126)	(2.1417)
Dwellings^2^ (*1000)	−0.0000	−0.0001**	0.0000	−0.0001**	0.0000	−0.0001**
	(−0.2207)	(−2.3719)	(0.0257)	(−2.3651)	(0.3577)	(−2.1093)
Merger year *t*	−0.0435	−0.0007	−0.0533*	−0.0071	−0.0245	−0.0435
	(−1.5583)	(−0.0255)	(−1.9528)	(−0.2622)	(−0.5109)	(−0.9856)
Merger year *t* − *1*	−0.0169	0.0399	−0.0213	0.0386	−0.0070	0.0041
	(−0.5959)	(1.5522)	(−0.7675)	(1.5299)	(−0.1525)	(0.1172)
Merger year *t* − *2*	−0.0233	−0.0190	−0.0269	−0.0158	−0.0117	−0.0393
	(−0.7944)	(−0.6233)	(−0.8592)	(−0.4849)	(−0.2735)	(−0.9980)
Merger year *t* – *3, …, T*	0.0201	0.0299	0.0181	0.0434	0.0091	−0.0052
	(0.5798)	(0.9794)	(0.4595)	(1.2843)	(0.1809)	(−0.1203)
Average age of housing stock^a^	−0.0142***	−0.0124***	−0.0101**	−0.0084	−0.0118***	−0.0113***
	(−3.5663)	(−3.2326)	(−2.0770)	(−1.5504)	(−3.2416)	(−3.0507)
Soil quality^a^	−0.1155	0.1225	−0.1859	−0.1529	0.1122	0.2406
	(−0.5872)	(0.6055)	(−0.6305)	(−0.5166)	(0.4953)	(1.0665)
Density^a^	0.0825***	0.0620**	0.0791***	0.0547*	0.0946***	0.0512
	(3.4495)	(2.3751)	(2.9332)	(1.7969)	(2.5904)	(1.5332)
Constant	−0.2139	−0.7225***	−0.3303	−0.6460*		
	(−0.9967)	(−2.9178)	(−1.1019)	(−1.7465)		
N	3912	3912	1701	1701	3135	3135
R-squared	0.1936	0.1305	0.2187	0.1408	0.1724	0.1262
Kleibergen-Paap rk Wald F statistic					28.70	28.70

Panel analysis 2002–2012. Fixed effects and year effects included. Robust *t* statistics (regressions 1–4) and *z* statistics (regressions 5–6) (based on clustered standard errors) between brackets

*** *p* < 0.01; ** *p* < 0.05, * *p* < 0.1

^a^As a bootstrap specification does not allow the model to control for exogenous characteristics, the efficiency scores cannot be corrected for differences in exogenous factors a priori. Therefore, these factors have to be included as control variables in the regression equation

Note that corporations can alter their scale in two ways: through organic growth (building, buying) and by merging. To disentangle these two components, we include both a variable measuring the scale level that has been reached through organic growth *(dwellings organic)* and a variable measuring the number of dwellings obtained by merging *(dwellings merger).* 16

Organic growth appears to have a positive impact on *pure technical efficiency* in the same year (regression 2). This impact is moderated by a negative lagged effect, however. This is probably a result of how we use the data. If a corporation builds dwellings at the end of year *t*, we perceive it as a scale increase in year *t*. Total costs in year *t* will probably increase only moderately, since in the first months of the year nothing happened. The net effect of organic growth on pure technical efficiency is still positive, however [0.1053–0.0744 = 0.0309 (or 3 percent for an increase in the number of dwellings by 1000)]. The effect of growth by merger is smaller (0.0131 or 1.3 percent) but significant.^17^ This supports the shake-up hypothesis. It is also consistent with the findings of Lupton and Kent-Smith (2012) that merging may be beneficial because it leads to a reconsideration of existing practices, thereby improving pure technical efficiency. According to regression (2), this does not only hold for merging but for organic growth as well.

Note that the effects are economically small: a scale increase of 1000 dwellings leads to an increase in pure technical efficiency of about 1.3–3 percent (minus the very small effect of the quadratic term). Such scale increases only occur with mergers. Organic growth deals with much smaller numbers.

The effect of merging *on total efficiency* (regression 1) is not significantly different from zero. This is not surprising, considering our earlier result that many corporations operate under diseconomies of scale. The net effect of organic growth (0.1070–0.1355 = −0.0285) is negative, however, indicating that the decrease in scale efficiency dominates the increase in pure technical efficiency. This indicates that organic growth is unfavourable from an efficiency point of view.

In short, growth—whether organic or by merger—seems to improve pure technical efficiency. However, it appears that increasing scale does not succeed in raising total productivity, because for many corporations, it reduces scale efficiency. This indicates the existence of a merger paradox.

### Robustness tests with DEA

The results from regressions (1) and (2) in Table 7 may be biased because the decision to merge is obviously not a random (or purely exogenous) process. It may depend upon many factors, one of which might be pre-merger efficiency. Similarly, organic growth may also be driven by initial efficiency. As a result, our control group includes corporations that may be incomparable because they did not merge.

The selection effect of merging can be mitigated by dropping the corporations that did not merge in our research period from the regressions. The control group then consists of corporations that merged, just like the treatment group, but in a different year. Regressions (3) and (4) in Table 7 give the results, which turn out to be very similar to regressions (1 and 2). Therefore, it appears that our results are not driven by a selection effect.

Concerning organic scale increases, the reverse causation problem may be dealt with by means of instrumental variables (IV) regression. We instrument the number and the squared number of dwellings by (1) the (first and second order) lagged number of dwellings, (2) the (first and second order) lagged number of dwellings, squared and (3) the number of dwellings that the subnational government is planning to facilitate in the region where the corporation is active. The latter variable is based on *De Nieuwe Kaart van Nederland*, a dataset comprising all housing projects that subnational governments are planning to implement. We presume that corporations operating in regions with such plans have a stronger incentive for organic growth than others. Also, we assume this variable is exogenous as it reflects decisions of subnational governments, not corporations.

Regressions (5) and (6) in Table 7 give the results of the IV-regression. According to regression (6), growth by merger still increases pure technical efficiency, but organic growth loses significance. This implies that the net effect of organic growth clearly is negative (regression (5)). The net effect of a merger on total efficiency remains insignificant, still indicating the existence of a merger paradox.

### Robustness check with SFA

The average (pure technical) efficiency scores from the stochastic frontier analysis are given in Table 8. Average inefficiency amounts to about 25–30 percent per year, which is higher than under DEA.

**Table 8:** Efficiency scores SFA

Year	*N*	Mean	SD	Min	Max
2001	525	0.69	0.08	0.38	0.97
2002	546	0.70	0.08	0.39	0.97
2003	531	0.70	0.08	0.40	0.97
2004	508	0.71	0.08	0.40	0.97
2005	494	0.71	0.08	0.41	0.97
2006	490	0.72	0.08	0.42	0.97
2007	453	0.72	0.07	0.54	0.96
2008	445	0.73	0.07	0.55	0.96
2009	426	0.73	0.07	0.55	0.97
2010	414	0.74	0.07	0.56	0.97
2011	388	0.75	0.07	0.57	0.97
2012	374	0.75	0.07	0.58	0.97

As noted, just as under DEA, SFA allows us to construct measures of total productivity change and decompositions into (1) pure technical efficiency change, (2) a scale effect and (3) technological change (see the “Appendix” for calculations). Table 9 provides the results.

**Table 9:** TFP-decompositions under SFA

Period	Pure efficiency change	Technological change	Scale Effect	Total factor productivity change
Merged corporations				
2001/2002	0.0085	−0.0549	−0.0618	−0.1083
2002/2003	0.0084	−0.0456	−0.0565	−0.0937
2003/2004	0.0083	−0.0398	−0.0676	−0.0991
2004/2005	0.0080	−0.0323	−0.0482	−0.0725
2005/2006	0.0078	−0.0234	−0.0464	−0.0620
2006/2007	0.0081	−0.0174	−0.036	−0.0453
2007/2008	0.0081	−0.0093	−0.0358	−0.0371
2008/2009	0.0075	−0.0032	−0.025	−0.0207
2009/2010	0.0072	0.0066	−0.0257	−0.0118
2010/2011	0.0069	0.011	−0.0203	−0.0024
2011/2012	0.0065	0.0177	−0.0106	0.0137
Unmerged corporations				
2001/2002	0.0088	−0.0586	0.0004	−0.0493
2002/2003	0.0086	−0.0519	0.0004	−0.043
2003/2004	0.0084	−0.0444	0.0000	−0.0361
2004/2005	0.0082	−0.0369	0.0000	−0.0287
2005/2006	0.008	−0.0295	0.0000	−0.0215
2006/2007	0.0078	−0.0218	0.0001	−0.0139
2007/2008	0.0076	−0.0147	0.0000	−0.0071
2008/2009	0.0074	−0.0076	−0.0002	−0.0003
2009/2010	0.0072	0.0000	−0.0002	0.0071
2010/2011	0.0071	0.0076	0.0000	0.0147
2011/2012	0.0069	0.0105	0.0000	0.0220

Several issues pop up. First, it appears that pure technical efficiency change is close to zero in most cases. This holds both for corporations that merged and for those that did not merge. That is, these figures fail to affirm the shake-up hypothesis that pure technical efficiency change is higher for institutions that merge. This conflicts with the DEA-results. Secondly, the scale effect turns out to be negative for corporations that have merged, affirming the notion that merging leads to increased diseconomies of scale. For corporations that did not merge, there was hardly any scale effect since their scale changed only marginally. So according to these results, mergers were unfavourable and we do not find a merger paradox. Note that in the final 2 years (2010/2011 and 2011/2012) the scale effect was very moderate, even for corporations that merged. So it seems that the scale issue may become less important over time. Secondly, total factor productivity change is negative in most years, but turns positive in the final few years. This may be a consequence of the increased public attention that the subject of efficiency has received in recent years as a result of the incidents (see also Sect. 6.1).

## Conclusion

This paper presents estimates of the efficiency of Dutch housing corporations and investigates the relationship between scale and efficiency. A data envelopment analysis indicates that the potential to improve pure technical efficiency is about 15 percent. If corporations would optimize their scale, a further gain of around 15 percent could be realized. Furthermore, total productivity failed to increase between 2002 and 2009. Thereafter, an upward trend seems to have set in.

The social housing sector has experienced many mergers throughout the years. We find that most housing corporations operate under diseconomies of scale, meaning that mergers could be undesirable. However, a merger might also have beneficial effects on pure technical efficiency, possibly because it forces parties to reconsider their existing practices and gives an opportunity to learn from each other. A data envelopment analysis confirms this shake-up hypothesis because a merger seems to have a positive effect on pure technical efficiency. However, a stochastic frontier analysis fails to replicate this result, indicating that the support for our shake-up hypothesis is not robust.

Furthermore, we argue that even if there would be such a positive effect, this should not be used as a justification to merge. Indeed, high technical efficiency should be attainable without merging as well. That is, no merger should be needed to optimize current processes. Ideally, decisions about changing the scale should be based upon the presence of (dis)economies of scale.

In order to improve our understanding of potential shake-up effects of mergers, case studies might be considered. Also, apart from mergers, many other factors may impact housing corporation efficiency, e.g. leadership, market power, organizational structure. Much work remains to be done.

## Appendix: data envelopment analysis

Data envelopment analysis was introduced by Charnes et al. (1978) who based their method on the ideas of Farrell (1957). The method constructs the best practice frontier of a group of decision making units (dmu’s) by solving a set of linear programming problems. This frontier gives all combinations of inputs and outputs that are relatively efficient. Consequently, every dmu is compared to this frontier to determine its efficiency. The inefficient dmu’s lie inside the frontier. The further away from the frontier, the less efficient a dmu is.

The linear programming problem in the input-oriented setting, following the notation of Coelli (1996), reads:

1 $$\begin{aligned} { \hbox{min} }_{{\theta_{i} ,\lambda }} \theta_{i} \hfill \\ s.t. \hfill \\ \varvec{X\lambda } \le \varvec{x}_{\varvec{i}} \theta_{i} \hfill \\ \varvec{Y\lambda } \ge \varvec{y}_{\varvec{i}} \hfill \\\varvec{\lambda}\ge 0 \hfill \\ \end{aligned}$$

Here *θ*_*i*_ denotes the efficiency score of dmu *i*, and $\varvec{x}_{\varvec{i}}$ and $\varvec{y}_{\varvec{i}}$ are, respectively, the input and output vectors of dmu *i*. $\varvec{X}$ and $\varvec{Y}$ are the input and output matrices for the entire set of dmu’s. Finally, $\varvec{\lambda}$ is a vector of weights to be determined in the optimization problem, so that $\varvec{X\lambda }$ and $\varvec{Y\lambda }$ are the weighted sum of, respectively, inputs and outputs of a ‘virtual dmu’. In the model, we thus search whether there exists a possibility to ‘defeat’ dmu *i*, by constructing a virtual dmu, being a linear combination of all existing dmu’s.

The virtual dmu needs to meet the requirements that it produces at least as many outputs and uses less input compared to dmu *i*. If we fail to construct a virtual dmu that meets these requirements, the efficiency score of dmu *i* obtains its maximum value of 1. The efficiency score *θ*_*i*_ thus reveals by how much total input of dmu *i* could decrease without decreasing output (‘measure of defeat’). The virtual dmu succeeds in producing the same amount of output as dmu *i* using only a fraction *θ*_*i*_ of inputs. Similarly, one could also choose an output orientation where the efficiency score can be interpreted as the percentage with which output could increase without increasing input.

Note that specification (1) does not impose any constraints on the weights of the virtual dmu. Therefore, an extra constraint on the weights might be introduced allowing for a variable returns to scale (vrs) technology:

2 $$\varvec{\lambda}{^{\prime }}1_{\varvec{N}} = 1$$

where $1_{\varvec{N}}$ is a vector of ones. Under this constraint, the virtual dmu has to be of the same size as dmu *i.*

### Non-discretionary inputs and exogenous variables

Some inputs may be non-discretionary (or fixed). These inputs are relevant factors in the production process in year *t* but cannot be influenced anymore during the production process (see Banker and Morey 1986). Denoting such variables by the vector $\varvec{q}_{\varvec{i}}$, we include as a constraint:

3 $$\varvec{Q\lambda } \le \varvec{q}_{\varvec{i}}$$

As noted in Sect. 4.2, closely related to the concept of fixed inputs is the idea that there may exist exogenous variables that influence efficiency scores. Several methods to take these exogenous variables into account are available. We follow an approach suggested by Ruggiero (1998), consisting of three steps. In the first step, an ordinary DEA is performed. Next, regression analysis is used to investigate which exogenous factors have an impact on efficiency.

4 $${\text{Efficiency}} = \beta_{0} + \mathop \sum \limits_{r = 1}^{R} \beta_{r} z_{r} + \varepsilon$$

where *z*_*r*_ (*r* = 1,…, *R*) are the relevant exogenous variables. In the second step, a variable *Z* is created that indicates to what extent a dmu is (dis)advantaged.

5 $$Z = \mathop \sum \limits_{r = 1}^{R} \beta_{r} z_{r}$$

A higher value of* Z* thus indicates a bigger advantage. In the third step, the DEA is repeated with the extra restriction:

6 $$\lambda_{j} = 0 \quad if \quad Z_{i} < Z_{j}$$

Due to this restriction, dmu *i* can only be compared with other dmu’s that are not exogenously advantaged relative to dmu *i.* In other words, advantaged dmu’s are being removed from the best practice frontier.^18^

### Malmquist indices

The Malmquist index (see Färe et al. 1994; Coelli 1996) is calculated by comparing two production points of a dmu while keeping the frontier fixed. In mathematical terms:

7 $$M^{\text{tfpch}} = \left[ {\left( {\frac{{{\text{TE}}_{t + 1,t}^{\text{crs}} }}{{{\text{TE}}_{t,t}^{\text{crs}} }}} \right) \left( {\frac{{{\text{TE}}_{t + 1,t + 1}^{\text{crs}} }}{{{\text{TE}}_{t,t + 1}^{\text{crs}} }}} \right)} \right]^{1/2}$$

where *M* ^tfpch^ is the total factor productivity change of a dmu and TE_*t*+1,*t*_^*crs*^ is the constant returns to scale efficiency score for a certain dmu where the input/output vector in period *t* + *1* is compared to the technology in period *t*. The index is thus the geometric mean of two measures of efficiency change (one relative to the frontier in year *t*, and the other relative to the frontier in year *t* + *1*).

Total factor productivity change can be written as the product of *M* ^techch^, a change in technology (i.e. the total shift of the frontier over time), and *M* ^effch^, the change in efficiency (the extent to which a dmu approaches the frontier) (Coelli 1996):

8a $$M^{\text{tfpch}} = M^{\text{techch}} *M^{\text{effch}}$$

Under a crs specification, *M* ^effch^ (i.e. approaching the crs-frontier) can be subdivided into pure technical efficiency change (*M* ^pech^; i.e. approaching the vrs-frontier) and scale efficiency change (*M* ^sech^; i.e. operating on a more efficient scale):

8b $$M^{\text{effch}} = M^{\text{pech}} *M^{\text{sech}}$$

In the example of Fig. 2, a movement from E to C resembles pure efficiency change and a movement from C to B indicates an increase in scale efficiency.

### Estimating the effects of mergers on scale efficiency and pure technical efficiency

In our analysis, we empirically investigate the effects of scale increases and mergers on efficiency. Because we are dealing with panel data, a dependent variable has to be constructed that can be compared over time. Therefore, we define efficiency in year *t* as the efficiency in year *t* − *1* multiplied by the relevant (bootstrapped) Malmquist index between *t* − *1* and *t*. For example, if total efficiency (Eff^tfp^) for a certain dmu is 0.5 in the first year and the Malmquist index (*M* ^tfpch^) equals 1.5 in the following year, our measure of efficiency in the second year equals 0.5*1.5 = 0.75.

We express efficiency in natural logarithms so that each year, the dependent variable changes with (the logarithm of) the Malmquist index. The dependent variables, respectively, total efficiency and pure technical efficiency thus read:

9a $${ \ln }\left( {{\text{Eff}}_{t}^{\text{tfp}} } \right) = { \ln }\left( {{\text{Eff}}_{t - 1}^{\text{tfp}} *M_{t - 1,t}^{\text{tfpch}} } \right) = { \ln }\left( {{\text{Eff}}_{t - 1}^{\text{tfp}} } \right) + { \ln }\left( {M_{t - 1,t}^{\text{tfpch}} } \right)$$

9b $${ \ln }\left( {{\text{Eff}}_{t}^{\text{pe}} } \right) = { \ln } \left( {{\text{Eff}}_{t - 1}^{\text{pe}} *M_{t - 1,t}^{\text{pech}} } \right) = { \ln } \left( {{\text{Eff}}_{t - 1}^{\text{pe}} } \right) + { \ln } \left( {M_{t - 1,t}^{\text{pech}} } \right)$$

### Stochastic frontier analysis

A stochastic frontier analysis econometrically estimates a production or cost function. We estimate the following translog cost function, using the random effects model of Battese and Coelli (1992):

10 $$\begin{aligned} { \ln }\left( C \right) & = \alpha_{0} + \mathop \sum \limits_{m = 1}^{M} \beta_{m} { \ln }\left( {Y_{m} } \right) + \mathop \sum \limits_{k = 1}^{K} \delta_{k} { \ln }\left( {Z_{k} } \right) + \frac{1}{2}\sum\limits_{m = 1}^{M} {\sum\limits_{m = 1}^{M} {\beta_{mm} { \ln }\left( {Y_{m} } \right){ \ln }\left( {Y_{m} } \right)} } \\ & \quad + \frac{1}{2}\sum\limits_{k = 1}^{K} {\sum\limits_{k = 1}^{K} {\delta_{kk} { \ln }\left( {Z_{k} } \right){ \ln }\left( {Z_{k} } \right)} } + \sum\limits_{m = 1}^{M} {\sum\limits_{k = 1}^{K} {\vartheta_{mk} { \ln }\left( {Y_{m} } \right)\ln \left( {Z_{k} } \right) + \varphi_{t} t} } \\ & \quad + \frac{1}{2}\varphi_{tt} t^{2} + \sum\limits_{m = 1}^{M} {\psi_{m} { \ln }\left( {Y_{m} } \right)t + v + u} \\ \end{aligned}$$

where *C* = costs;* Y*_*m*_ = output (*m* = 1, …,* M*);* Z*_k_ = exogenous factor (*k* = 1, …,* K*);* v* = random error term (normal distribution);* u* = inefficiency term (truncated normal distribution); *t* = time.

Note that we do not include input prices because these are not available. We correct our cost measures for inflation in order to make them comparable over the years.^19^ This is more appropriate than explicitly including price indices in the model, since these indices would suffer from multicollinearity with *t*.

Also, for estimation, we have standardized the data to the mean beforehand (i.e. for each variable *C*, *Y* or *Z* we divide each observation by its mean in 2012). Standardization has the advantage that the estimated parameters can be interpreted as elasticities at the sample mean (Ollinger et al. 2000). Also, standardization reduces the problem of multicollinearity between linear, squared and cross terms (Tovar et al. 2007).

The results of the estimation are given in Table 10.

**Table 10:** Cost function estimates (translog cost function)

Variable	Coefficient	Variable	Coefficient	Variable	Coefficient
*Y*_1_	0.1176***	*Z*_1_ * *Z*_1_	0.2232***	*Z*_3_ * *Y*_1_	−0.0819
	(3.9548)		(4.8887)		(−1.2199)
*Y*_2_	0.9596***	*Z*_2_ * *Z*_2_	0.0160	*Z*_3_ * *Y*_2_	0.0768
	(29.4856)		(0.5322)		(0.9933)
*Y*_3_	0.4012**	*Z*_3_ * *Z*_3_	0.2358	*Z*_3_ * *Y*_3_	0.5972
	(2.3142)		(0.3592)		(1.5504)
*Z*_1_	0.4365***	*Z*_1_ * *Z*_2_	0.0334	*t*	0.0607***
	(8.6970)		(0.7020)		(8.0025)
*Z*_2_	−0.0137	*Z*_1_ * *Z*_3_	−1.2380***	*t* * *t*	−0.0072***
	(−0.7535)		(−4.2929)		(−8.4641)
*Z*_3_	0.0966	*Z*_2_ * *Z*_3_	0.1404	*Y*_1_ * *t*	−0.0053***
	(1.1731)		(1.5398)		(−2.6800)
*Y*_1_ * *Y*_1_	0.0108	*Z*_1_ * *Y*_1_	0.0176	*Y*_2_ * *t*	−0.0000
	(1.3510)		(0.6659)		(−0.0021)
*Y*_2_ * *Y*_2_	0.0425***	*Z*_1_ * *Y*_2_	0.0114	*Y*_3_ * *t*	−0.0226
	(3.5749)		(0.3935)		(−1.4070)
*Y*_3_ * *Y*_3_	−0.0576	*Z*_1_ * *Y*_3_	0.0633	$Dummy Y_{1} = 0$	0.1861
	(−0.1706)		(0.3704)		(1.5162)
*Y*_1_ * *Y*_2_	−0.0254***	*Z*_2_ * *Y*_1_	0.0202*	Constant	−0.5096***
	(−3.7178)		(1.9444)		(−8.3279)
*Y*_1_ * *Y*_3_	0.0479	*Z*_2_ * *Y*_2_	−0.0218*	Observations	5594
	(0.6409)		(−1.6486)	Years	2001–2012
*Y*_2_ * *Y*_3_	0.0153	*Z*_2_ * *Y*_3_	0.1025		
	(0.1863)		(1.1688)		

Results based on the random effects model of Battese and Coelli (1992) with a truncated normal distribution of the efficiency term

*z* statistics in parentheses (based on clustered standard errors)

*** *p* < 0.01; ** *p* < 0.05; * *p* < 0.1

### Decomposing total factor productivity change

The coefficients of the cost function, together with the efficiency scores, may be used to decompose total factor productivity change into (1) the change in efficiency, (2) technological change and (3) a scale effect (see, e.g. Orea 2002; Abdul-Majid et al. 2011). In general, we have:

11 $${\text{Tfp}}\,{\text{change}} = {\text{efficiency}}\, {\text{change}} + {\text{technological}}\, {\text{change}} + {\text{scale}}\, {\text{effect}}$$

This is calculated as follows:

12a $$\ln \left( {\frac{{{\rm TFP}_{it + 1} }}{{{\rm TFP}_{it} }}} \right) = \ln \left( {\frac{{{\rm TE}_{it + 1} }}{{{\rm TE}_{it} }}} \right) - \frac{1}{2}\left[ {\frac{{\partial \ln \left( {C_{it + 1} } \right)}}{\partial t} + \frac{{\partial \ln \left( {C_{it} } \right)}}{\partial t}} \right] + \frac{1}{2}\mathop \sum \limits_{m = 1}^{M} [\left( {{\rm SF}_{it + 1} \varepsilon_{{\rm mit} + 1} + {\rm SF}_{it} \varepsilon_{\rm mit} } \right)(y_{{\rm mit} + 1} - y_{\rm mit} )]$$

where:

12b $$\varepsilon_{mit} = \frac{{\partial { \ln }\left( {C_{it} } \right)}}{{\partial { \ln }\left( {y_{mit} } \right)}}$$

12c $$SF_{it} = \left( {\mathop \sum \limits_{m = 1}^{M} 1 - \varepsilon_{mit} } \right)/\mathop \sum \limits_{m = 1}^{M} \varepsilon_{mit}$$

### Dealing with unbalanced panel data

Note that we have an unbalanced panel because of the mergers. We handle this by taking the corporation classification of the first year (2001) as a starting point. If two corporations (A and B) merge to one corporation (AB) between 2001 and 2002, we thus still have two separate observations in 2002 (that is, corporation AB now returns twice in the dataset). So, in effect, we estimate both the efficiency effects of A growing towards AB and of B growing towards AB. Note that in this case, we would have ‘identical twins’ in our dataset from 2003 onwards (as AB pops up two times each year). Therefore, we exclude one of these ‘identical twins’ from our regression from 2003 onwards.

## Footnotes

1 The terms housing association or housing society are sometimes used as well. In judicial terms, corporations are either foundations or associations. In this paper, we use the term (housing) corporation.

2 Source: CorpoData and Statistics Netherlands.

3 On the other hand, increasing scale through merging may reduce competition, which may increase organizational slack (CPB 2013).

4 Note that since July 1, 2015, a new housing Act has been implemented (replacing the BBSH), aiming to strengthen the ties between the government and corporations and improve supervision. During 2001–2012, the BBSH was standing legislation. However, although monitored, the goal attainment was not enforced by the Dutch government.

5 If we were to include ‘municipal housing companies’, who provided part of the social housing in the twentieth century, the total number was 1152 in 1985. Nowadays, all social housing is in the hands of corporations. Source: Hakfoort et al. (2002).

6 Because some data are missing and we eliminate outliers, not all corporations can be included in the analyses, however.

7 In fact, exogenous characteristics are often controlled for by treating them as nondiscretionary inputs. Such a method is, however, outperformed by the method of Ruggiero (1998).

8 Other decompositions of the Malmquist indices are possible (see Simar and Wilson (1998a, b)) for example).

9 We have not explicitly included the number of new developments, since this already pops up in the (increased) number of inhabited dwellings. As long as a new dwelling remains uninhabited, we argue that it should not be counted as output since it does not fulfil requirement 3.

10 Note that one may argue that a distinction between types of dwellings (single-family units, multiple family units) might also be relevant. Including too many outputs might lead to problems of overidentication, however, meaning that many corporations will be labelled as efficient by definition (Nunamaker 1985).

11 Part of the Netherlands consists of sinking marshland, which results in relatively high maintenance costs.

12 Different thresholds do not lead to changes in the main conclusions (results not shown).

13 Note that because DEA defines scale by means of all outputs and inputs, not by number of dwellings, it is possible that both economies and diseconomies of scale occur within the group of corporations with 1001–2500 dwellings (and in other groups). Also, not all corporations with more than 2500 dwellings operate under diseconomies of scale. In other words: one cannot simply point out an optimal number of dwellings.

14 Simar and Wilson (1998a, 1999) note that efficiency scores estimated by means of DEA may be biased because they are derived using finite data samples. The true production possibility frontier is not observed; changing the data would change the results. Statistical estimation needs replication of the data-generating process. Therefore, we use the bootstrap procedure of Simar & Wilson (1999), replicating the data-generating process in order to correct for potential biases and obtain confidence intervals (see also Arjomandi et al. 2011; Gitto and Mancuso 2012).

15 We do not include a column with the effects of mergers on scale efficiency, as this would not be very informative. Indeed, if two corporations that already operate under diseconomies scale merge, diseconomies will increase by definition (and scale efficiency would decrease). Note, however, that the regression of mergers on total efficiency also includes a component of scale efficiency. This column is included in order to show the net effect of mergers on total factor productivity.

16 These variables are constructed as follows. In the first year of measurement (2002), *dwellings organic* simply equals the total number of dwellings of each corporation and *dwellings merger* is zero. *Dwellings organic* increases or decreases throughout the years if the corporation alters its housing stock by building, demolishing, buying or selling. *Dwellings merger* increases by the extent of a merger, if a merger occurs. By definition, the sum of the two variables equals the total housing stock of the corporation.

17 Because the lagged variable of *dwellings merger* is not significant, we dropped this variable from the regression.
